# Safety Profile and Adverse Event Management in Flexible Endoscopic Treatment of Zenker’s Diverticulum: A Systematic Review

**DOI:** 10.3390/medicina62102029

**Published:** 2026-10-20

**Authors:** Noemi Gualandi, Giuliano Francesco Bonura, Giulia Bertoncini, Gabriella Frassanito, Tommaso Gabbani, Marcello Maida, Davide Bona, Pablo Cortegoso Valdivia, Paola Soriani, Mauro Manno

**Affiliations:** 1 Gastroenterology and Digestive Endoscopy Unit, AUSL Modena, 41012 Carpi, Italy; n.gualandi@ausl.mo.it (N.G.); g.bonura@ausl.mo.it (G.F.B.); gi.bertoncini@ausl.mo.it (G.B.); g.frassanito@ausl.mo.it (G.F.); t.gabbani@ausl.mo.it (T.G.); p.soriani@ausl.mo.it (P.S.); 2 Department of Medicine and Surgery, University of Enna ‘Kore’, 94100 Sicily, Italy; 3 Gastroenterology, Umberto I Hospital, Enna 94100, Italy; 4 Division of Surgery, Department of Biomedical Sciences for Health, IRCCS Galeazzi Hospital, University of Milan, 20157 Milan, Italy; 5 Gastroenterology and Endoscopy Unit, University Hospital of Parma, University of Parma, 43126 Parma, Italy; cortegosopablo@yahoo.it; 6 Department of Clinical Research, University of Southern Denmark, 5230 Odense, Denmark; 7 Surgical Research Unit, Odense University Hospital, 5000 Odense, Denmark

**Keywords:** Zenker’s diverticulum, flexible endoscopic septotomy, Z-POEM, adverse events, systematic review

## Abstract

*Background and Objectives*: Flexible endoscopic septal division (FESD) and third-space techniques represent first-line treatments for symptomatic Zenker’s diverticulum (ZD). While technical and clinical success rates are well established, data regarding adverse events (AEs) and their clinical management remain fragmented in recent literature. This systematic review aimed to evaluate the incidence, typology, and therapeutic strategies for AEs associated with flexible endoscopic ZD treatments. *Materials and Methods*: A systematic search of PubMed/MEDLINE, Embase, and Scopus was conducted up to July 2026 according to PRISMA guidelines. Studies reporting adult patients undergoing flexible endoscopic interventions for ZD reporting data on complication rates and management were included. Methodological quality was evaluated using the MINORS index. *Results:* Sixty studies were evaluated, comprising 53 cohort series (64 cohorts, 3172 patients, 3266 procedures: 2119 FESD and 1147 third-space procedures) for pooled quantitative incidence estimation, and 7 single-case reports retained for qualitative complication mapping. Bleeding occurred in 4.2% of cases (5.9% in FESD vs. 1.1% in third-space techniques), with most events occurring intraoperatively and resolving entirely through endoscopic hemostasis. Perforation occurred in 3.2% of procedures (3.9% in FESD vs. 1.8% in third-space series), while mucosal leaks were reported in 0.6% (0.2% in FESD vs. 1.5% in third-space). Severe infectious sequelae, such as abscesses (0.2%) and acute mediastinitis (0.1%), were rare. Conservative measures and endoscopic salvage modalities successfully treated transmural defects, with surgical conversion required in only three perforation cases (<0.1%). Isolated subcutaneous emphysema or pneumomediastinum occurred in 1.3% of cases and resolved uneventfully under conservative observation. Direct procedure-related mortality was 0.03% (1/3266). *Conclusions*: Flexible endoscopic management of ZD demonstrates a high safety profile with minimal procedural mortality. Complication management has shifted from surgical rescue to conservative protocols and versatile endoscopic salvage. Standardized AE grading and ongoing randomized trials are required to refine procedural selection.

## 1. Introduction

Zenker’s diverticulum (ZD) represents an acquired posterior hypopharyngeal mucosal and submucosal herniation through Killian’s triangle, an area of anatomical weakness bounded by the oblique fibers of the inferior pharyngeal constrictor and the horizontal fibers of the cricopharyngeal (CP) muscles [1,2]. ZD is the most common esophageal diverticulum, with an estimated prevalence ranging between 0.01 and 0.11%, predominantly affecting elderly individuals in their seventh and eighth decades [2]. The functional relevance of ZD in this geriatric population has grown in parallel with extending life expectancy.

Pathophysiologically, ZD is not a primary esophageal disorder but a pulsion pseudodiverticulum arising from structural and neuromuscular CP dysfunction. Progressive fibrosis and decreased tissue compliance lead to impaired coordination between hypopharyngeal bolus propulsion and sphincter relaxation during deglutition, causing elevated intraluminal pressure that drives gradual outpouching of the posterior hypopharyngeal wall through Killian’s triangle [3]. Progressive oropharyngeal dysphagia dominates clinical presentation in 80% to 90% of symptomatic cases. Associated manifestations include regurgitation, chronic cough, halitosis, and aspiration pneumonia, severely degrading quality of life and precipitating frailty and malnutrition [4,5].

Based on clinical symptoms, the diagnosis and the characterization of ZD is confirmed by barium swallow radiography with fluoroscopy [2], alongside with upper esophagogastroduodenoscopy (EGD) to rule out concomitant pathologies. However, conventional contrast radiography carries diagnostic limitations: while demonstrating pouch size and dynamic retention, it cannot characterize transmural wall architecture, differentiate muscular thickness, or resolve prior submucosal fibrosis and inflammatory scarring. Treatment aims at complete CP myotomy, restoring a common esophageal lumen [3,6], and it is recommended for all symptomatic patients, irrespective of the diverticulum’s size [2].

Over the past two decades, high-definition instruments and novel accessories have established flexible endoscopic septum division (FESD) as first-line therapy over historical surgical approaches [2]. Recent technical modifications, such as endoscopic myectomy of the CP muscle, have been conceived to allow a wider removal of the segment of muscle [7].

Despite these advantages, FESD remains technically demanding. Visual constraints limit myotomy depth and carry an inherent risk of transmural injury, often preventing complete CP muscle division in small (<2 cm) or heavily fibrotic diverticula [8].

To overcome these limitations, third-space endoscopy emerged with Zenker’s peroral endoscopic myotomy (Z-POEM), introduced by Hernández-Mondragón et al. in 2018 [9]. By creating a controlled submucosal tunnel, Z-POEM provides direct visualization for precise, complete cricopharyngeal myotomy while preserving the overlying mucosa. Recently, a wide array of third-space endoscopic techniques and hybrid approaches have rapidly emerged to ensure a patient-tailored approach. These techniques can be divided in two groups based on the procedural paradigm: mucosa-preserving tunneling techniques (including standard Z-POEM, single-tunnel Z-POEM, and peroral endoscopic septotomy [POES]), which isolate the dissection plane within a submucosal tunnel and achieve closure of the mucosal entry via clips; and incision-guided, unroofing, or non-tunneling modifications (including open Z-POEM [OZ-POEM], Z-POEM with mucosotomy, tunnelling-free Z-POEM, peroral endoscopic diverticulotomy [POED], submucosal injection-guided septomyotomy [SING], transverse incision and longitudinal septostomy [TILS]), and non-injection, non-tunneling Z-POEM (NiZ-POEM) [5,10]. In the latter approaches, the septal mucosa is deliberately incised, resected, or unroofed to facilitate exposure and prevent recurrent diverticular pouching, relying on secondary-intention re-epithelialization rather than primary closure of the mucosal barrier.

Nevertheless, current literature has disproportionately prioritized clinical/technical success and recurrence rate, while data on adverse events (AEs) remain fragmented. This systematic review aggregates the safety profile of flexible endoscopic treatments, to provide a clinical overview on the definition and management of complications in ZD.

## 2. Materials and Methods

### 2.1. Data Sources and Search Strategy

A systematic literature search was performed on PubMed/MEDLINE, Scopus and Embase databases from 1 January 2006 to 31 July 2026. The search strategy combined Medical Subject Headings (MeSH) and free-text terms related to “Zenker’s Diverticulum” and “Adverse Events”. A manual review of the reference list of included studies followed the electronic search. No automated publication-type or language filters were applied within the initial database search strings. The complete search strings are available in the Supplementary Materials S1. This systematic review was conducted in accordance with the PRISMA checklist requirements.

### 2.2. Inclusion and Exclusion Criteria

Studies were evaluated eligible for inclusion if they met the following criteria: (1) adult patients (older than 18 years old) diagnosed with symptomatic ZD requiring interventional treatment; (2) treatment performed via flexible endoscopic treatment (FESD, Z-POEM, POES and hybrid techniques); (3) availability of detailed data regarding AEs and treatment strategies. We intentionally included case reports, case series, retrospective/prospective cohorts to ensure a comprehensive analysis of rare complications and their management. Single-case reports and small descriptive series were deliberately retained to map exceptionally rare complication phenotypes and novel salvage maneuvers, while being segregated from quantitative incidence pooling to prevent selection bias.

Exclusion criteria were: (1) pediatric populations or animal models; (2) non-flexible endoscopic approaches, such as open surgery or using rigid transoral endoscopes; (3) stapling, laser-assisted septotomy, magnetic devices or purely otolaryngological/surgical techniques were excluded (to guarantee homogeneity in safety profiles and treatment of complications); (4) studies published prior to 2006 (to reflect contemporary clinical practice and current technological standards); (5) duplicate or overlapping patient cohorts from the same institution covering identical recruitment windows (only the most recent or largest cohort was retained); (6) non-English publications.

### 2.3. Screening of References

After excluding duplicates, two authors independently screened references (N.G. and G.F.B.). Each author screened all references (title and abstract) according to the inclusion and exclusion criteria. In case of discrepancy, the article was included for full-text evaluation. This approach was repeated on included references with both authors assessing the full text. Primary and full-text screening was conducted in Rayyan [11].

### 2.4. Data Extraction

Data were extracted following the Preferred Reporting Items for Systematic Reviews and Meta-Analyses (PRISMA) [12]. Data were independently extracted by three reviewers (N.G., G.F., G.B.) into a standardized spreadsheet. One reviewer (N.G.) controlled all the final data.

Variables collected included: study design; clinical and demographic features of patients included; diverticular size; prophylactic clipping/antibiotic therapy; type of endoscopic procedure performed; type of knife/accessories (such as transparent cap, diverticuloscope) used; AE rates, type, severity and temporal classification of AEs; specific therapeutic approach and management of complications; 3-months-mortality and the mean/median follow-up.

For continuous data, results were reported as mean or median, depending on how they were presented in the original article.

### 2.5. Quality Assessment

Included studies underwent an assessment of methodological quality and risk of bias by three independent reviewers (N.G., G.F. and G.B.) using the Methodological Index for Non-Randomized Studies (MINORS) [13] assessment tool. MINORS was chosen over alternative tools due to the prevalent nature of ZD literature, mainly consisting of retrospective, non-randomized cohorts and case-matched series. For studies with a non-comparative design, the 12-item Non-Comparative MINORS checklist was applied. Each item was scored from 0 to 2 (0 = not reported, 1 = reported but inadequate, 2 = reported and adequate), yielding an ideal global score of 16. For comparative studies, the 8 additional items for comparative evaluations were included, leading to an ideal global score of 24 Supplementary Materials S2.

Single-case reports (included for qualitative synthesis and rare complication mapping) were excluded from the numerical MINORS scoring to avoid methodological distortion. Any discrepancies in scoring among the three reviewers were resolved through discussion and consensus.

### 2.6. Definition of Outcomes

The main endpoints were the safety profile of flexible endoscopic treatments for ZD, defined by the incidence, type, and management of AEs.

AE rates were calculated considering the total number of distinct procedures as denominator. AEs were categorized according to clinical manifestation: bleeding, perforations/mucosal leaks, subcutaneous emphysema, mediastinitis, post-procedural fever, post-procedural pain, and other related events.

Due to the lack of a universally defined temporal framework for complications and the heterogeneous definitions in included studies, AEs were defined as:

- Intra-procedural bleeding: persistent hemorrhage requiring any form of hemostatic intervention.
- Post-procedural bleeding: hemorrhage requiring endoscopic re-evaluation and/or hospital readmission.
- Intra-procedural perforation: endoscopic visualization of perivisceral structures through a full-thickness parietal wall defect.
- Post-procedural perforation: cervical subcutaneous emphysema confirmed by extraluminal barium extravasation on fluoroscopic swallow radiography, or free mediastinal air associated with a fluid collection.

Isolated subcutaneous emphysema or pneumomediastinum without radiologically confirmed contrast extravasation or fluid collections was not classified as perforation. Instead, suspected perforations or micro-perforations reported without further specification were recorded as AEs. Secondary infectious sequelae, such as mediastinitis and abscesses, were analyzed as discrete AEs rather than pooled with the primary AE rate.

Severity of complications was graded according to the American Society for Gastrointestinal Endoscopy (ASGE) Lexicon and the Appraisal of Guidelines for Research & Evaluation (AGREE) criteria [14,15]. All data regarding therapeutic strategies were systematically extracted and synthesized.

### 2.7. Data Synthesis

A formal quantitative meta-analysis was precluded due to clinical and methodological heterogeneity across the included literature. Specifically, studies exhibited divergent reporting thresholds for intraoperative bleeding, widely disparate follow-up durations (ranging from short hospital stays to multi-year assessments), lack of standardized time-to-event data, and non-uniform adoption of formal AE grading systems. Consequently, data were narratively synthesized by grouping outcomes according to procedural modality and AE subtype. To provide descriptive epidemiological estimates, cumulative incidence rates were calculated by pooling the total number of documented procedures as the common denominator. Nevertheless, because these calculations derive predominantly from retrospective and observational series, the resulting aggregate rates must be interpreted cautiously in light of inherent selection and documentation biases.

Systematic pooling of several baseline parameters was unfeasible due to heterogeneity of data reporting: patient age and diverticulum dimensions were reported inconsistently as mean or median values, preventing overall aggregate estimations. Moreover, baseline American Society of Anesthesiologists (ASA) score and concurrent antithrombotic/antiplatelets therapy were rarely documented. Although cohorts often distinguished naïve patients from recurrent cases, complication rates were rarely stratified by prior intervention, precluding a dedicated sub-analysis. A large language model (Google Gemini 1.5 Flash) was used solely for language refinement and organizational support.

## 3. Results

**Figure 1:**
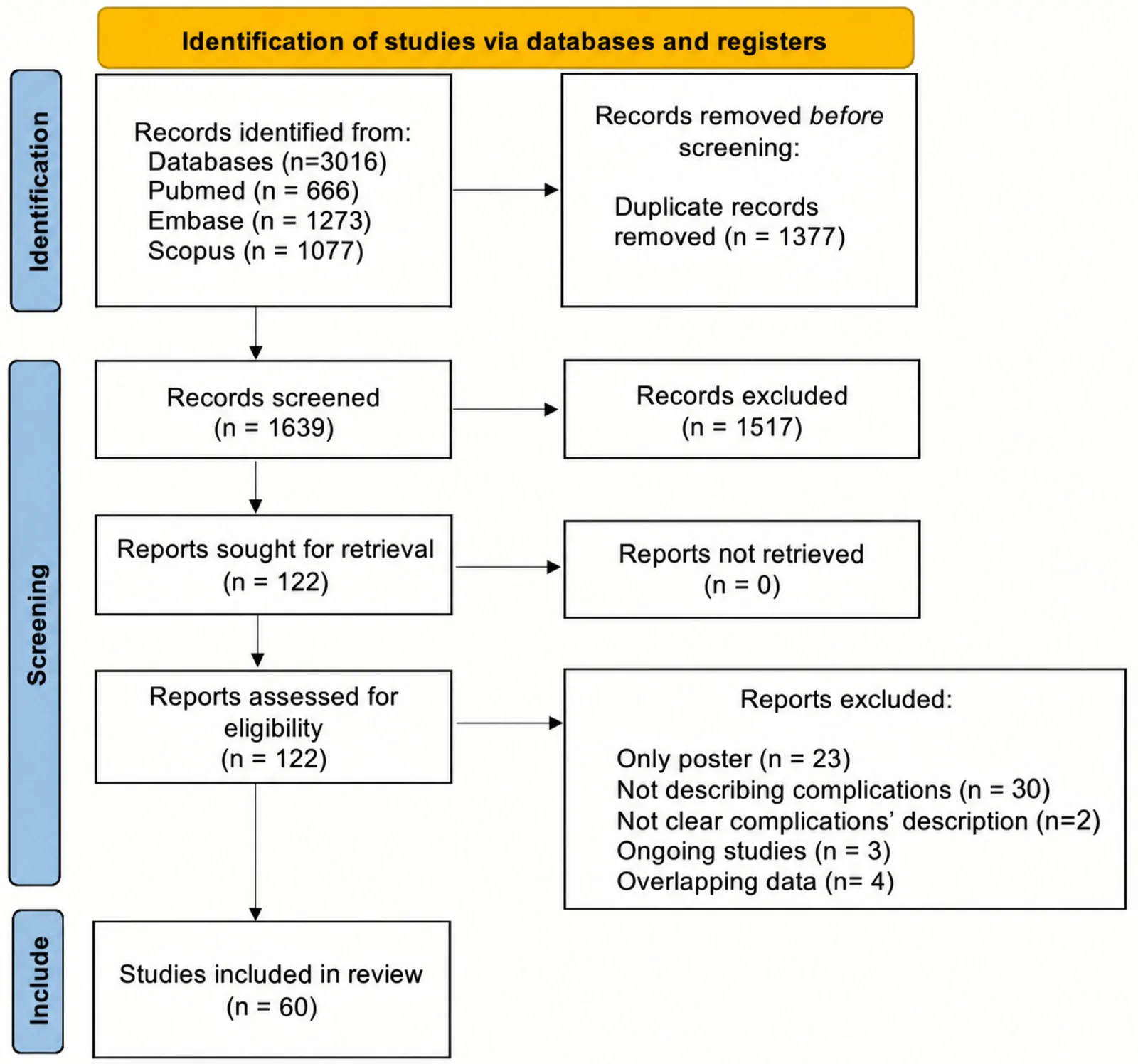
Prisma Flowchart.

Overall, 3016 references were identified in the initial search. After exclusion of duplicates, abstract screening excluded 1517 records, leaving 122 references for full-text reading. Sixty (*n* = 60) studies (35 retrospective observational studies, 4 prospective cohorts, 7 case reports and 14 comparative analyses) met the inclusion criteria (Figure 1) [7,16,17,18,19,20,21,22,23,24,25,26,27,28,29,30,31,32,33,34,35,36,37,38,39,40,41,42,43,44,45,46,47,48,49,50,51,52,53,54,55,56,57,58,59,60,61,62,63,64,65,66,67,68,69,70,71,72,73,74].

Across these 60 publications, 78 distinct procedural arms were initially identified. Six surgical or rigid endoscopic septotomy arms were excluded as per protocol. Furthermore, to prevent patient double-counting across multicenter registries with overlapping recruitment, the Z-POEM cohort from Norton et al. 2024 (*n* = 45) was excluded from pooled quantitative synthesis, retaining only the non-overlapping FESD cohort from that study alongside the expanded Z-POEM series from Norton et al. 2025 (*n* = 61) [50,70]. Finally, 7 single-case reports were reserved exclusively for qualitative complication and rescue mapping [16,24,35,37,39,47,49]. Consequently, 64 discrete cohorts across 53 observational studies were retained for quantitative AE pooling. The detailed characteristics of the included studies are summarized in Table 1.

For quantitative incidence pooling, 3266 distinct procedures were evaluated across 64 cohorts (3172 patients): 39 cohorts underwent flexible endoscopic septotomy (2026 patients, 2119 procedures) and 25 cohorts underwent third-space techniques (1146 patients, 1147 procedures). Overall, 1129 patients were female (35.6%) and 1786 were male (56.3%), with sex not reported in 257 patients (8.1%). Direct procedure-related mortality was documented in only one case across the analyzed cohorts (1/3266, 0.03%), secondary to a fatal intraoperative cardiac arrest during a third-space intervention [45]. One additional late death was described by Norton et al. [50] occurring four months post-procedure due to aspiration pneumonia following clinical recurrence; as this was secondary to treatment failure rather than procedural trauma, it was adjudicated as disease-related mortality.

Overall and specific incidence of AEs are displayed in Table 2.

### 3.1. Bleeding

**Figure 2:**
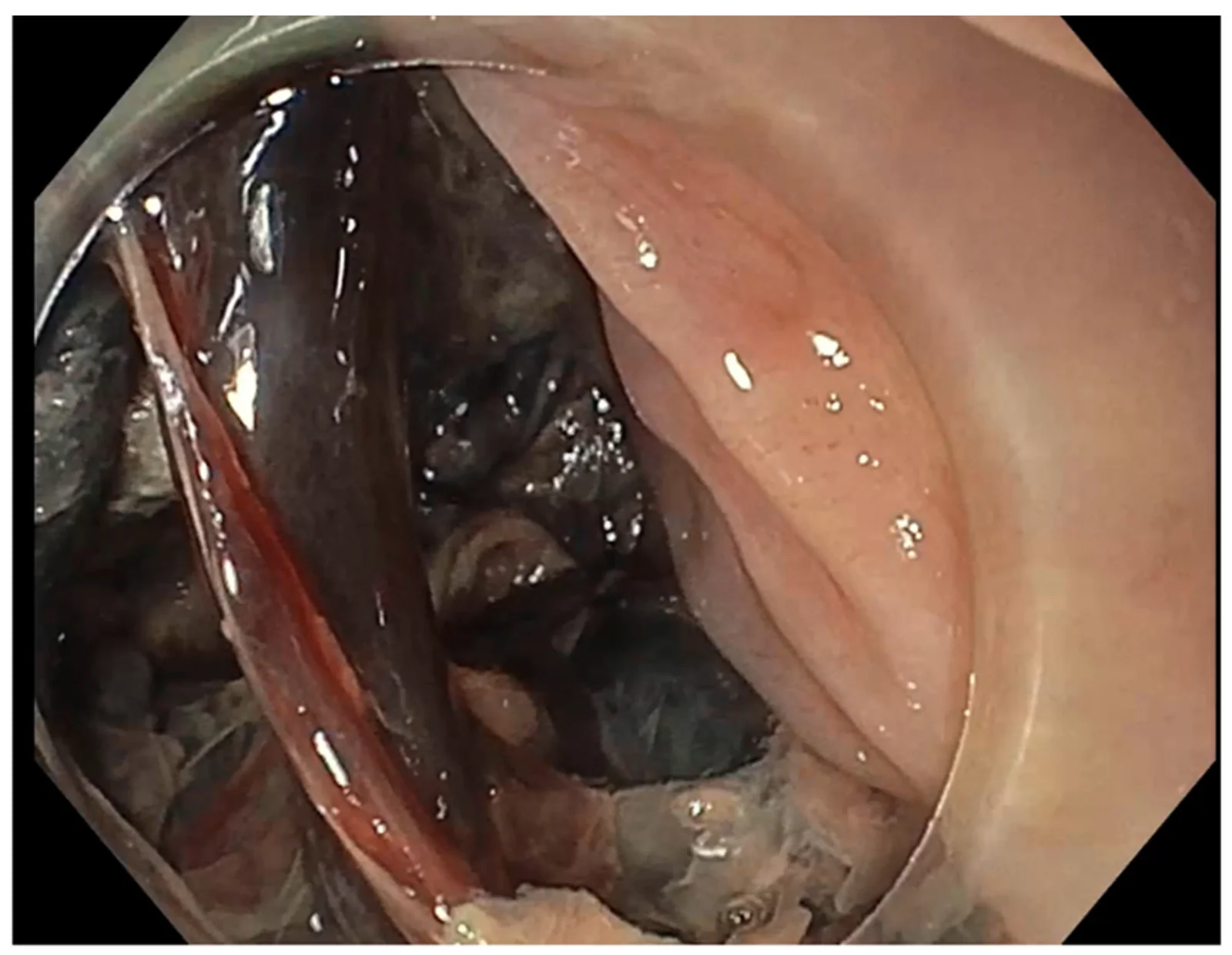
Post-procedural bleeding.

Bleeding represents the most frequent AE across the analyzed literature, occurring in 4.2% of overall cases (137 events/3266 procedures, in 37/64 cohorts) (Figure 2). The clinical spectrum ranged from self-limiting intra-procedural oozing to delayed arterial hemorrhage, with therapeutic strategies increasingly centered on endoscopic management rather than surgical conversion.

#### 3.1.1. Etiology and Risk Factors

Most bleeding events, reported across the analyzed studies, were considered as minor/mild complications and occurred intra-procedurally (almost 76%), due to minor vessel transection during septal division. While previous large retrospective series [18,26] routinely documented these bleedings effectively managed during the index procedure, recent clinical trials [68,74] frequently omitted minor intra-procedural bleeding from formal AE registries, potentially underestimating its true incidence.

Early series utilizing standard needle-knives reported frequent intra-procedural bleeding [21] compromising endoscopic visualization and requiring auxiliary hemostatic maneuvers such as thermal coagulation, endo-clip placement, or injection therapy. Nevertheless, the progressive integration of specialized submucosal dissection knives (e.g., Triangle Tip Knife J, Hybrid Knife) and dedicated scissor-type devices (e.g., Stag Beetle Knife, Clutch Cutter) has improved intraoperative safety profiles. Continuous mucosal lavage instantly clears the operative field while dedicated electrosurgical devices allow a targeted tissue dissection alongside real-time vessel pre-coagulation, without frequent auxiliary device exchanges, thus suppressing rates of post-procedural hemorrhage [27,69,72].

Post-procedural hemorrhage occurs less frequently than intraoperative oozing but it carries substantial clinical relevance, typically manifesting within 24 to 48 h as hematemesis, melena, or an abrupt hemoglobin decline requiring urgent re-endoscopy [22]. The identification of definite clinical predictors remains challenging; in our review, evaluating the impact of ongoing antiplatelet/anticoagulant regimens and baseline ASA scores was precluded due to incomplete reporting across available cohorts.

#### 3.1.2. Third-Space Techniques vs. FESD

Bleeding was documented in 5.9% of FESD procedures (124/2119) compared to 1.1% (13/1147) in third-space techniques.

This numerical divergence suggests a superior hemostatic control in submucosal tunneling and hybrid procedures due to direct vessel visualization within the submucosal space. However, critical temporal and methodological confounders warrant careful interpretation. FESD cohorts span a long timeline dating back to 2007 [21] reflecting older device generations and lower thresholds for reporting minor intra-procedural oozing. In contrast, third-space and hybrid series represent a contemporary era from 2020 onward [54,70], benefiting from modern electrosurgical units and evolving complication reporting standards.

#### 3.1.3. Management

Endoscopic intervention provided definitive hemostasis across all documented bleeding episodes, with no patient requiring emergency surgical conversion for hemorrhage.

Hemostasis during intra-procedural bleeding was achieved primarily through diluted adrenaline injection, contact thermal coagulation with the dissecting knife (e.g., ClutchCutter, Stag Beetle Knife, Triangle Tip Knife J) [27,66,69], dedicated hemostatic forceps (e.g., Coagrasper, Hemostat), probe and argon plasma coagulation, or topical hemostatic matrix application [37]. Mechanical hemostasis was utilized selectively via through-the-scope (TTS) or over-the-scope clips (OTSC).

Post-operative bleeding episodes were successfully managed by urgent repeat EGD with targeted thermal coagulation [22] or clip application over the culprit vessel [52,56]. Topical hemostatic agents and supportive red-blood-cell transfusions were administered when clinically indicated [68].

To mitigate the bleeding incidence, a rigorous perioperative optimization is warranted. Correct withholding and resumption of antiplatelet and anticoagulant therapies according to the European Society of Gastrointestinal Endoscopy (ESGE) guidelines [75], baseline risk stratification of the patient assessment (according to ASA score) and a proactive endoscopic hemostasis of visible vessels before withdrawal is fundamental.

In this context, the identification of procedural protective measures remains an open debate, particularly regarding prophylactic clipping at the septotomy base. Foundational series, such as the one published by Huberty et al. (*n* = 150) [25] and de la Morena Madrigal et al. [26], established routine clip placement as a standard maneuver to prevent bleeding. Conversely, more recent multicenter analyses by Steinbrück et al. [76] and Mittal et al. [68] demonstrate on multivariable regression that prophylactic clipping neither independently prevents delayed hemorrhage nor enhances long-term clinical success. In fact, prolonged clip retention at the myotomy base can stimulate local tissue granulation and progressive fibrosis, providing an anatomic substrate for delayed symptom recurrence [77].

Contemporary practice reserves clipping predominantly for active oozing or high-risk mucosal defects, aligning with standardized AE grading systems such as the ASGE or AGREE lexicon.

### 3.2. Perforations, Leakage, and Related Sequelae

Perforations represent the second most frequent AE evaluated in our review, with an overall incidence of 3.2% (103 cases/3266 procedures, in 41/64 cohorts). Esophageal leakage is less common, with a 0.6% incidence (21 cases/3266 procedures). Their incidence and management strategies have undergone a significant shift in recent years, moving from surgical rescue therapy toward endoscopic management.

#### 3.2.1. Etiology and Risk Factors

Perforations primarily arise when identifying the distal border of the septum proves difficult. As highlighted by Costamagna et al. [22], extending the incision too deeply to ensure complete myotomy has a higher risk of entering the mediastinal space. Mittal et al. [68] and Klinger et al. [65] observed that small pouches (<2 cm) or prominent CP bars reduce safety margins due to the paucity of a clear dividing wall. Large diverticula (>5 cm) also increase perforation risks due to the extended dissection needed [22]. Device choice and careful depth control are therefore essential to avoid tissue injury.

New dissecting instruments may reduce the risk of perforation during myotomy. Dedicated devices with insulated tips (e.g., Insulated Tip Knife) provide enhanced stability during caudal incision and minimize inadvertent deep muscular damage [78], whereas scissor-type instruments (e.g., Stag Beetle Knife and Clutch Cutter) limit deep thermal and mechanical injury through their non-conductive exterior surfaces and controlled grasping action [68].

However, procedural trauma is not limited to electrosurgical cutting. Accessory devices such as the soft diverticuloscope improve septal exposure but carry mechanical risks, particularly in elderly and frail patients with marked tissue fragility. Insertion-related trauma can cause extensive hypopharyngeal perforation, as highlighted in reports of pyriform sinus perforations [17,24].

Postoperative airway management also influences wall integrity. Immediate use of positive pressure ventilation, such as continuous positive airway pressure (CPAP), can reopen small mucosal defects and drive extraluminal leaks, making temporary suspension of CPAP prudent during early tissue healing [16,23].

Finally, to mitigate the severity of gas-related complications, ESGE guidelines recommend the routine use of carbon dioxide (CO_2_) due to its rapid tissue resorption compared to nitrogen-rich room air. This rapid diffusion reduces the incidence and severity of pneumomediastinum and subcutaneous emphysema.

#### 3.2.2. Infectious Sequelae: Cervicomediastinal Abscess and Mediastinitis

Cervicomediastinal abscess and acute mediastinitis represent the most severe sequelae of perforation or leakage, carrying a high risk of sepsis.

Within our cohort analysis, abscesses occurred in 4 cases from FESD group (0.2%, 4/2119) and 2 cases from third-space endoscopy series (0.2%, 2/1147), while mediastinitis was explicitly documented in 2 cases in FESD (0.1%, 2/2119) [22,50] and 2 within the third-space endoscopy (0.2%, 2/1147) [53,65].

This distribution indicates that infectious complications can occur regardless of the specific technical modality, being driven primarily by local microbial contamination and baseline patient frailty.

#### 3.2.3. Third-Space Techniques vs. FESD

The precise temporal distribution of perforations cannot be unequivocally defined for all analyzed cohorts, because several studies report AEs in an aggregated or non-specified manner. A comparative evaluation demonstrates a perforation incidence of 3.9% in the FESD group (82/2119 procedures) versus 1.8% in the third-space group (21/1147 procedures). Conversely, mucosal leaks were predominantly observed in third-space techniques (1.5%, 17/1147 procedures) compared to FESD (0.2%, 4/2119 procedures). The combined rate of transmural defect AEs (perforation and leak) was 4.1% in FESD (86/2119) versus 3.3% in third-space interventions (38/1147).

The higher incidence of perforation observed in the FESD group relies on anatomical, procedural, and historical factors: the absence of reliable distal landmarks during top-down full-thickness transection [79], the absence of an intact mucosal barrier, and historically the free-hand cutting technique, further compounded by a substantial cohort disparity and longer reporting history (2119 procedures vs. 1147 procedures in third-space series).

#### 3.2.4. Management

**Figure 3:**
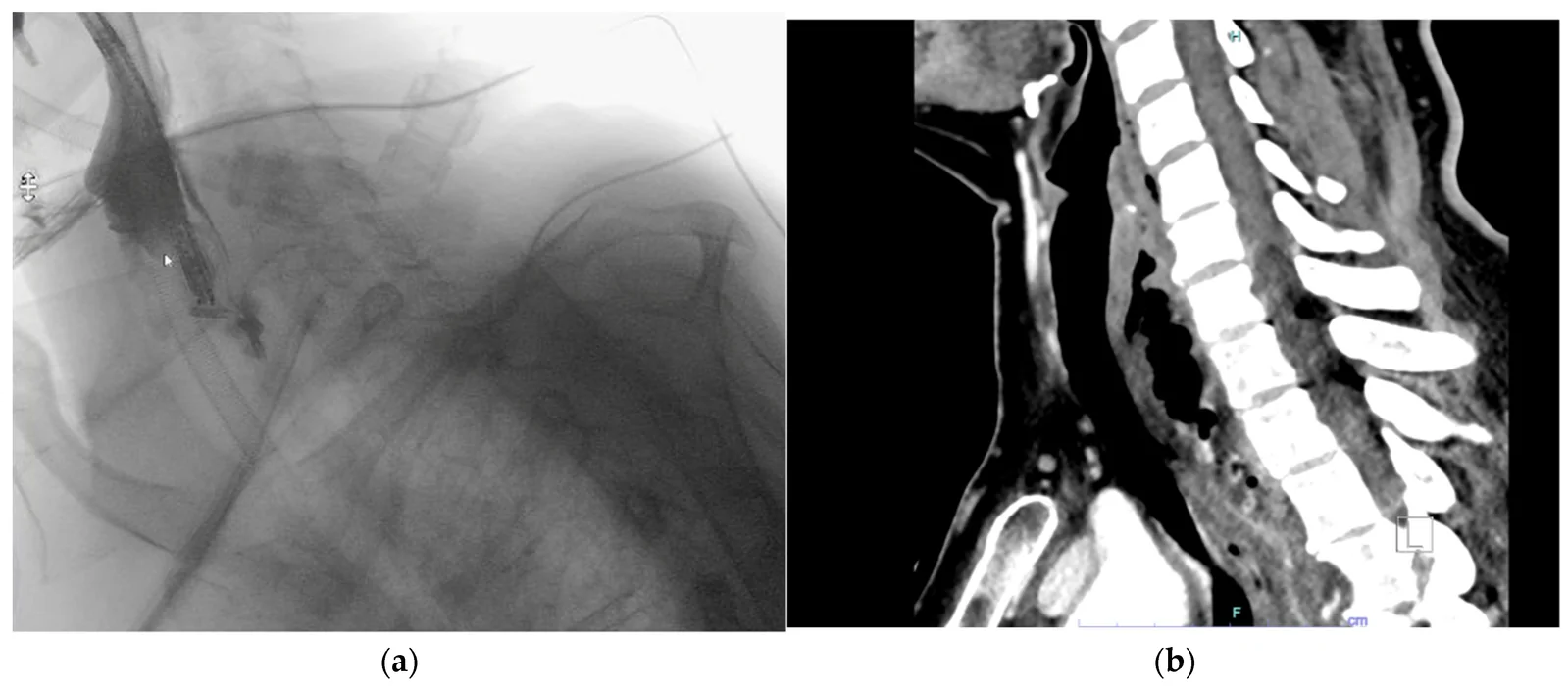
(**a**) Post-procedural perforation, fluoroscopic contrast extravasation. (**b**) Post-procedural perforation, evidence of extraluminal free air in the retroesophageal and prevertebral spaces.

Prompt detection remains critical to optimizing clinical outcomes. Perforation is typically identified intra-procedurally through direct endoscopic visualization or suspected postoperatively upon the onset of persistent chest pain, odynophagia, cervical crepitus, or fever. Diagnostic confirmation relies on radiographic imaging, including contrast-enhanced esophagograms or computed tomography (Figure 3a,b).

Post-operative diagnostic pathways remain varied across centers; while some protocols incorporate routine contrast swallow studies to systematically exclude subclinical leaks prior to oral refeeding [42], other algorithms adopt a symptom-triggered imaging approach reserved exclusively for patients exhibiting clinical deterioration [58].

Conservative management represents the first-line therapeutic standard for most iatrogenic perforations and localized extraluminal complications. In stable patients, initial treatment centers on strict nil-per-os status, broad-spectrum intravenous antibiotic coverage, and targeted enteral or parenteral nutritional support [21,25,50].

Endoscopic approaches are considered the primary interventional rescue strategy, using TTS clips, OTSCs, self-expanding metal stents, glue, rubber band techniques [24], fibrin sealant [35], and other endoscopic suturing devices [49] to seal transmural defects. Even in case of large fluid collections endoscopic interventions are now the primary treatment modality. Hoppo et al. [47] successfully managed complex post-Z-POEM mediastinal collections using endoscopic longitudinal mucosal dissection to convert a closed abscess cavity into a drained space. Daza Castro et al. [39], and Alsaad et al. [16] used an effective endoscopic vacuum therapy (EVT) to drain the collection.

Open surgical conversion remains exceptional. Only three cases of esophageal perforation [30,55,65] required subsequent surgical closure. On the other hand, 7 on 10 cases of persistent collections were treated with surgical or percutaneous interventions [20,22,45,46,50,51].

The routine prophylactic clipping of septotomy bottom remains an area of active discussion. ESGE guidelines [2] leave the decision to the operator preference or to clinical necessity, meta-analytic data from Ishaq et al. [4] show no significant reduction in postoperative AEs. Evaluating the true protective efficacy of baseline prophylactic in FESD cohorts was precluded by the retrospective nature of most cohorts and the paucity of granular data.

While FESD often leaves the septal defect open, Z-POEM requires mucosal closure. Fayyaz et al. [23] challenge this necessity by introducing the OZ-POEM technique, which involves extensive mucosotomy and leaving the mucosal flap open to heal by secondary intent. Their data, supported by the comparison with clip-based Z-POEM, suggest that this approach may be equally safe and potentially more effective in preventing late recurrences due to the eradication of the redundant mucosal flap.

Data on antibiotic administration remain heterogeneous, with only a subset of studies analyzed reporting pre-, peri- or post-operative coverage using penicillins, cephalosporins, or ciprofloxacin. Current ESGE guidelines advise against the use of routine prophylactic antibiotics before septotomy, because of the absence of a significant reduction in AEs, result confirmed also in other large cohort of patients, Mittal et al. (*n* = 161) [68] and Steinbrück et al. (*n* = 384) [76].

In our data extraction, the majority of studies reporting postoperative abscesses either omitted prophylactic antibiotics or did not report their use, with Aden et al. representing the sole exception [56].

### 3.3. Subcutaneous Emphysema and Pneumomediastinum

Subcutaneous emphysema and pneumomediastinum are distinctive clinical and radiographic phenomena observed following ZD endoscopic treatment. In our analysis the overall incidence was 1.3%.

#### 3.3.1. Etiology and Risk Factors

Contemporary literature distinguishes overt transmural perforations from benign, pressure-induced gas tracking through dissected tissue planes. Even if, some author [50] still defines perforation as the development of subcutaneous emphysema.

These phenomena are driven by CO_2_ or air insufflation during the myotomy of the CP muscular bridge, the elevation of intraluminal pressure leads translocation of gas along the fascial planes of the neck and superior mediastinum, through micro-openings of tissue [31].

Current literature reports an incidence of post-procedural emphysema ranging from 2.3% to 23% [31,76], largely depending on diagnostic scrutiny and insufflation parameters, particularly the choice of CO_2_ over room air. Budnicka et al. [19] reported a case of extensive emphysema and pneumomediastinum requiring intensive care monitoring and prolonged intubation, which was attributed to the simultaneous administration of CO_2_ and air.

Our database analysis revealed a similar incidence of emphysema between the two group, 1.1% in FESD (24/2119) and 1.7% in third space cohort (19/1147).

#### 3.3.2. Management

In the absence of extraluminal contrast extravasation, collections, systemic inflammatory response or hemodynamic instability, a conservative approach is usually chosen, consisting of short-term fasting (enteral o parenteral nutrition), intravenous antibiotic therapy, without requiring secondary interventions [27,29,31].

### 3.4. Non-Bleeding, Non-Perforation Adverse Events

Beyond bleeding and perforation, endoscopic management of ZD carries broader cardiopulmonary and other systemic risks, considering the high prevalence of elderly, comorbid population undergoing these procedures.

#### 3.4.1. Aspiration Pneumonia and Respiratory Complications

Aspiration pneumonia is both a primary preoperative indication for ZD intervention and a potential post-operative complication. According to our results, pneumonia occurred after 0.5% of procedures (17/3266; 0.4% in FESD vs. 0.7% in third-space techniques), in line with previous studies, Huberty et al. [25] reporting post-operative aspiration pneumonia in 0.7% of patients, while Juin et al. [62] (*n* = 19) documented a 10% overall medical complication rate, including one case of aspiration pneumonia related to anesthesia and extubation.

The use of soft diverticuloscope during myotomy may protects the airway from aspiration in non-intubated patients [25].

In the cohort by Norton et al. one fragile patient died due to aspiration pneumonia four months after a failed procedure, adjudicated as disease-related mortality [50], while Kahaleh et al. [63] mentioned one episode of bilateral pneumothorax during Z-POEM secondary to gas insufflation within the submucosal tunnel and subsequent communication with the pleural space.

#### 3.4.2. Other Adverse Events

Transient post-procedural nausea/vomiting, dysphagia, odynophagia, retrosternal chest pain, and low-grade fever are common minor or moderate events. These are related to local inflammatory response, often spontaneously resolved or managed conservatively with short-term analgesics and observation [26,59,66].

Cardiovascular events, such as transient arrhythmias [61,62], brady/tachycardia [32,63,66] are occasionally observed in response to the physiological stress of anesthesia in elderly patients [55].

Norton et al. [70] reported a case of persistent diarrhea requiring hospital readmission due to severe dehydration secondary to postoperative refeeding syndrome.

## 4. Discussion

Over the last two decades, flexible endoscopic modalities have progressively superseded open surgical repair and rigid endoscopic diverticulotomy for symptomatic ZD [80]. Open surgery entails significant morbidity (up to 11%) [5,81] and an estimated mortality of 1% to 2% [79], while rigid stapling carries a nearly 9% technical failure rate due to severe anatomical constraints, including micrognathia and restricted cervical spine mobility [5,79]. By contrast, FESD and third-space techniques achieve reproducible clinical success rates exceeding 90% with negligible procedural mortality (only 1 direct procedure-related death over 3266 procedures, 0.03% in our pooled cohort) [4,34]. This marked reduction in procedural morbidity, combined with accelerated functional recovery and minimized infection risk in geriatric populations, establishes the flexible endoscopic route as the front-line therapeutic standard, as endorsed by current ESGE guidelines [2].

In this systematic review of 3266 endoscopic procedures across 64 cohorts, flexible endoscopic treatments exhibited an overall robust safety profile with minimal procedural mortality, with the vast majority of AEs managed endoscopically and conservatively.

Bleeding and wall breach (perforation or leak) constituted the most prevalent AEs, displaying distinct procedural and temporal patterns. Hemorrhage occurred in 4.2% of cases overall (137/3266), with an evident numerical discrepancy between FESD (5.9%, 124/2119) and third-space tunneling techniques (1.1%, 13/1147). Rather than demonstrating an inherent physiological risk, this disparity reflects chronological reporting bias and technological iteration. Early FESD cohorts frequently recorded minor, self-limiting intra-procedural oozing managed as formal AEs. Conversely, contemporary third-space series omit minor intraoperative bleeding from complication logs. From a technical standpoint, modern electrosurgical platforms, high-pressure integrated water-jet knives, and rotatable scissor-type devices permit pre-emptive vessel coagulation before tissue cutting, drastically minimizing clinically relevant delayed hemorrhage.

Leakage and perforation display distinct procedural patterns, with spatial vectors of dissection dictating the perforation and leakage profile. FESD relies on a top-down, non-tunneled division of the septal bridge without precise distal boundaries. Once dissection approaches the base of the diverticulum, distinguishing the deep boundary of the CP muscle from the loose retroesophageal and mediastinal adventitia is technically challenging, explaining the higher rate of primary intraoperative transmural defects [79]. Conversely, tunneling techniques preserve an intact overlying mucosal flap. In Z-POEM, the primary failure mode is delayed mucosal leakage resulting from closure clip dehiscence or thermal injury to the mucosal flap (1.5% in Z-POEM vs. 0.2% in FESD cohort). This mechanical difference also explains why third-space modifications that intentionally abandon mucosal closure (e.g., OZ-POEM or SING) exhibit different healing kinetics, relying on secondary-intention re-epithelialization rather than mechanical mucosal integrity.

Small diverticula (<2 cm), prominent CP bar [68] or large pouches (>4 cm) [22] represent the main risk factors for transmural injury and perforation, as submucosal fibrosis and prior failed interventions [54,70]. To overcome this limit, advanced endoscopists frequently employ a “hybrid” approach to safely navigate scarred tissue [74].

Severe infectious sequelae, such as abscess (0.2% in both FESD and third-space cohorts) and acute mediastinitis (0.1% overall), remained rare. Notably, the therapeutic management of extraluminal collections has shifted away from open surgical re-exploration (required in only 6 cases across the entire database). Contemporary salvage algorithms favor multimodal endoscopic rescue, including TTS and OTS clipping, dedicated upper esophageal stents, and EVT to promote active cavity collapse and secondary intention healing [16,39]. Respiratory AEs, predominantly aspiration pneumonia (0.5% overall), underline the intrinsic frailty of the target geriatric population. In fact, the frequent malnutrition and multiple comorbidities of these patients significantly worsen clinical outcomes when procedural complications arise [26].

Procedural environment and airway management constitute fundamental determinants of clinical safety. Although conventional FESD can be performed under conscious sedation, general anesthesia with endotracheal intubation is strongly recommended, especially in contemporary practice, particularly during third-space tunneling septotomies [79]. Tracheal intubation definitively isolates the airway from regurgitated pouch contents, pre-procedural fasting and comprehensive pouch clearance prior to dissection [58] is mandatory, while providing immediate ventilatory support in case of complications [19]. Concurrently, executing myotomies within a fluoroscopy-equipped interventional suite facilitates real-time contrast extravasation assessment and immediate salvage maneuvers. Routine CO_2_ insufflation remains obligatory over room air to ensure rapid spontaneous resorption of benign subcutaneous emphysema or isolated pneumomediastinum (1.3% overall incidence) within 24–48 h. Notably, the vast majority of included studies omitted explicit descriptions of intraoperative airway protection and radiological setup, identifying a clear gap in peri-interventional documentation standards that need to be implemented in future trials.

Comparative safety between conventional septotomy and third-space modalities must be interpreted through within-study comparative cohorts rather than crude pooled sums across observational literature, which aligns with recent evidence [82]. Specifically, the meta-analysis by Delgado et al. [83] demonstrates no statistically significant differences between third-space techniques and FESD regarding overall AEs (13.94% vs. 10.24%, *p* = 0.73), major complications (6.59% vs. 3.68%, *p* = 0.61), esophageal perforation (4.77% vs. 3.90%, *p* = 0.89), or bleeding (2.88% vs 3.4%, *p* = 0.45). These findings align with pooled data from Singh et al. [84], suggesting comparable safety outcomes across both endoscopic treatment modalities. However, those pooled analyses aggregated diverse single-arm and comparative series without dissecting the procedural mechanics of complications or detailing specific rescue pathways.

Furthermore, existing literature is constrained by numerous variables: operator learning curves and inter-center procedural volumes are rarely disclosed, despite being recognized determinants of perforation risk during initial third-space adoption. Similarly, a degree of publication bias remains unavoidable, as single-center fatal events or severe complications are less likely to be reported for publication.

Several methodological limitations must be acknowledged. First, the imbalance between FESD cohorts (dating back to the 2000s) and third-space techniques (predominantly reported after 2020) introduces temporal bias regarding technology evolution and data reporting. Second, substantial heterogeneity in AE definitions and in the adoption of standardized grading systems (ASGE or AGREE criteria only in 41 of 72 cohorts) precluded formal quantitative meta-analysis, restricting synthesis to pooled descriptive rates. Third, clinical confounders (e.g., antiplatelet/anticoagulant regimens, ASA scores, diverticulum size, naïve vs. retreated patients) were sparsely documented, preventing stratified sub-analyses on patient-specific risk factors for AE development. Finally, the predominant reliance on retrospective series and the inclusion of single-case reports to capture rare sequelae exposes our results to documentation, reporting, and publication biases.

## 5. Conclusions

Flexible endoscopic septotomy and third-space techniques provide a favorable safety profile for symptomatic ZD, with negligible procedural mortality across more than 3200 documented procedures. The observed numerical differences in AE rates between conventional septotomy and submucosal tunneling reflect reporting evolution and technological iterations rather than true technical inferiority. This progress pattern is equally evident in complication management, decisively shifted from open surgical revision toward conservative stabilization and endoscopic salvage: active bleeding is effectively managed via targeted electrosurgical coagulation or mechanical clipping, while perforations and localized leaks are resolved using clips, endoscopic suturing, and EVT. Mandatory CO_2_ insufflation prevents severe barotrauma and facilitates rapid gas resorption.

Widespread implementation of standardized grading systems is required across future registries. Prospective randomized data from ongoing comparative studies, such as the ZIPPY trial (NCT04514042), will be critical to definitively establish tailored indications and compare long-term efficacy and safety between flexible septotomy and third-space modalities.

## Figures and Tables

**Table 1:** Characteristics of included studies.

Author, Year of Publication [Ref]	Study Design	Naïve/Retreated Patients (*n*)	Procedure/Patients (*n*)	F/M (*n*)	Technique	Total Rate of AEs %, (*n*)	Type of AEs (*n*)	Grade (AGREE or ASGE Lexicon)	AEs Management	Endoscopic Treatment	Follow-Up (Months)
Alsaad A. et al., 2024 [16]	Case Report	1/0	1/1	1/0	Z-POEM	N/A	Perforation (1), abscess (1)	Major	Endoscopic	OTSC, EVT	-
Azzolini F. et al., 2026 [17]	Multicenter retrospective cohort series	102/0	102/102	31/71	SING	2.9 (3)	Bleeding (2), perforation (1)	IIIa	-	-	15 (median)
Brueckner J. et al., 2016 [18]	Retrospective single-center cohort series	41/5	46/46	21/25	FESD	8.7 (4)	Bleeding (3), perforation (1)	-	Endoscopic	Bleeding: clip TTS	39 (mean)
Budnicka A. et al., 2021 [19]	Multicenter retrospective cohort series	20/2	22/22	8/14	Z-POEM	13.6 (3)	Emphysema (3)	Mild and Moderate	Conservative	No	8.5 (median)
Case D. J. et al., 2010 [20]	Retrospective single-center cohort series	18/4	22/22	9/13	FESD	50 (12)	Bleeding (5), perforation (6), abscess (1)	-	Surgery for drainage	Bleeding: injective hemostasis; perforation: clip TTS	12.7 (mean)
Costamagna G. et al., 2007 Cohort A [21]	Comparative retrospective single-center cohort series	28/0	28/28	10/18	FESD	32 (9)	Bleeding (4), perforation (5)	-	Endoscopic	Bleeding: thermal hemostasis; perforation: clip TTS	36 (median)
Costamagna G. et al., 2007 Cohort B [21]		11/0	11/11	6/5	FESD	0	-	-	-	-	6.5 (median)
Costamagna G. et al., 2016 [22]	Multicenter retrospective cohort series	89/0	89/89	36/53	FESD	3.4 (3)	Bleeding (1), perforation (2)	-	Surgery for drainage	Bleeding: thermal hemostasis (forceps)	32 (median)
Fayyaz F. et al., 2025 [23]	Multicenter retrospective cohort series	50/3	53/53	16/37	Z-POEM	3.8 (2)	Perforation (1), esophageal ulcer (1)	Mild and 1 Severe	Endoscopic	EVT	6 (median)
Frazzoni L. et al., 2019 [24]	Case Report	1/0	1/1	1/0	FESD	N/A	Perforation (1)	-	Endoscopic	clip TTS- clips-and-rubber band technique	1
Huberty V. et al., 2013 [25]	Retrospective single-center cohort series	142/8	150/150	54/96	FESD	2.2 (4)	Perforation (3), emphysema (1)	Mild and Moderate	Conservative	No	43 (median)
de la Morena Madrigal E.J. et al., 2016 [26]	Retrospective single-center cohort series	62/2	64/64	18/46	FESD	39 (25)	Bleeding (21), perforation (2), chest pain and fever (3)	Mild and 1 Severe	Endoscopic	Bleeding: thermal hemostasis; perforation: clip TTS	31 (mean)
Pang M. et al., 2019 Cohort A [7]	Comparative retrospective single-center cohort series	36/8	44/44	15/29	FESD	11.4 (5)	Bleeding (2), perforation (3)	-	Endoscopic	Bleeding: thermal, and injective hemostasis; perforation: stent	10.7 (median)
Pang M. et al., 2019 Cohort B [7]	-	10/10	20/20	6/14	FESD	10 (2)	Bleeding (2)	-	Endoscopic	Bleeding: thermal, and injective hemostasis	8.9 (median)
Rath T. et al., 2018 Cohort A [27]	Comparative prospective single-center vs. retrospective cohort series	NR	6/6	3/3	FESD	16.6 (1)	Bleeding (1)	Minor	Endoscopic	Bleeding: thermal hemostasis (Clutch Cutter)	12 (mean)
Rath T. et al., 2018 Cohort B [27]	-	NR	15/15	6/9	FESD	73 (11)	Bleeding (7), emphysema (3), perforation (1)	Mild and 1 Severe	Endoscopic	Bleeding: clip TTS, thermal hemostasis; perforation: clip TTS	-
Repici A. et al., 2021 [28]	Retrospective single-center cohort series	256/0	256/256	70/186	FESD	3.5 (9)	Bleeding (1), perforation (5), dysphagia (1), pharyngodynia (1), pneumonia (1)	Moderate and 1 Severe	Surgery for perforation	Perforation: clip TTS	65.1 (mean)
Repici A. et al., 2010 [29]	Prospective single-center consecutive case series	32/0	32/32	9/23	FESD	6.25 (2)	Bleeding (1), emphysema (1)	Minor and Moderate	Endoscopic	Bleeding: thermal hemostasis (APC)	23.9 (mean)
Schoeman S. et al., 2023 [30]	Retrospective single-center cohort series	70/73	240/193	78/115	FESD	7.1 (17)	Bleeding (6), perforation (6), delirium (1), thoracic pain (3), vomiting (1)	Moderate and 1 Severe	Surgery for perforation	Perforation: clip TTS	-
Vogelsang A. et al., 2007 [31]	Retrospective single-center cohort series	31/0	31/31	13/18	FESD	26.2 (8)	Bleeding (1), emphysema (7)	Minor	Endoscopic	Bleeding: thermal hemostasis (APC)	26 (median)
Ward M.A. et al., 2023 [32]	Multicenter retrospective cohort series	NR	40/40	15/25	Z-POEM	7.5 (3)	Perforation (1), bradycardia (1), Clostridium difficile colitis (1)	Mild and moderate	Endoscopic	Perforation: clip TTS	12 (mean)
Yang J. et al., 2020 [33]	Multicenter retrospective cohort series	75/11	75/75	33/42	Z-POEM	6.7 (5)	Bleeding (1), perforation (5)	Moderate and 1 Severe	Endoscopic	Perforation: clip TTS, cyanoacrylate glue	9.6 (median)
Zhang L.Y. et al., 2023 [34]	Multicenter retrospective cohort series	69/14	83/83	31/52	Z-POEM	4.8 (4)	Perforation (2), infective symptoms (2)	Mild and moderate	Endoscopic	-	12.2 (median)
Alkmos M. F. et al., 2025 [35]	Case Report	1/0	1/1	1/0	Z-POEM	N/A	Perforation (1)	-	Endoscopic	Perforation: APC and Fibrin Sealant	1
Almario J. A. et al., 2025 [36]	Multicenter retrospective cohort study	36/0	36/36	11/25	Z-POEM	2.8 (1)	Perforation (1)	Moderate	Endoscopic	Perforation: clip TTS	6.4 (median)
Boustani C. et al., 2026 [37]	Case Report	NR	1/1	NR	FESD	N/A	Bleeding (1)	-	Endoscopic	Bleeding: hemostatic matrix	NR
Calavas L. et al., 2021 [38]	Comparative retrospective multicenter cohort study	44/5	53/49	19/30	FESD	26.4 (14)	Bleeding (2), perforation (3), emphysema (5), fever (4)	-	Endoscopic	Bleeding: thermal hemostasis	41 (mean)
Daza Castro E. M. et al., 2023 [39]	Case Report	1/0	1/1	0/1	Z-POEM	N/A	Perforation (1), abscess (1)	-	Endoscopic	Perforation: Jakson Pratt drain, EVT	1
Ciuc D. et al., 2018 [40]	Comparative retrospective multicenter cohort study	29/0	29/29	NR	FESD	31 (9)	Bleeding (3), thoracic pain and fever (6)	-	Endoscopic	Bleeding: clip TTS and thermal hemostasis (Hemostat)	12 (mean)
Bresteau B. et al., 2020 [41]	Retrospective single-center cohort series	56/21	77/77	23/54	FESD	3.9 (3)	Pneumomediastinum (2), pneumonia (1)	Moderate and 1 Severe	Conservative	No	23 (mean)
Desai P.N. et al., 2020 [42]	Retrospective single-center cohort series	16/0	16/16	6/10	Z-POEM	18.8 (3)	Pneumomediastinum (2), bleeding (1)	Mild	Endoscopic	Bleeding: thermal hemostasis (Coagrasper)	18 (mean)
Al Ghamdi S.S et al., 2021 Cohort A [43]	Comparative retrospective multicenter cohort study	94/25	119/119	52/67	Z-POEM	16.8 (20)	Bleeding (3), mucosal tears (4), leaks (4), hematoma (1), emphysema (5), pneumonia (3)	Mild and moderate	Endoscopic	Mucosal tears: clip; leak: cyanoacrylate glue, stent	9.3 (mean)
Al Ghamdi S.S et al., 2021 Cohort B [43]	-	72/14	86/86	38/48	FESD	2.3 (2)	Bleeding (1), perforations (1)	Mild and moderate	-	-	8.6 (mean)
Gomez-Outomuro A. et al., 2019 [44]	Retrospective single-center cohort series	15/1	16/16	6/10	FESD	25% (4)	Perforation (1), thoracic pain (2), fever (3)	Mild and moderate	Conservative	No	9.2 (median)
Gupta V. et al., 2025 [45]	Retrospective single-center cohort series	54/3	57/57	33/24	Z-POEM	7 (4)	Edema-odynophagia (1), diarrhea (1), leak (1), cardiac arrest (1) **^§^**	II-IIIb-V	Surgery for drainage	No	9 (median)
Halland M. et al., 2016 [46]	Retrospective single-center cohort series	40/12	58/52	28/24	FESD	31 (18)	Bleeding (7), perforation (11), abscess (1)	Moderate and Severe	Surgery for drainage	Bleeding: thermal and injective hemostasis; perforation: stent	21 (median)
Hoppo T. et al., 2025 [47]	Case Report	1/0	1/1	1/0	Z-POEM	N/A	Perforation (1), abscess (1)	-	Endoscopic	Mucosal dissection	-
Ishaq S. et al., 2020 [48]	Retrospective single-center cohort series	36/29	65/65	39/26	FESD	6.2 (4)	Bleeding (2), hypoxia (2)	Minor	Endoscopic	Bleeding: endoscopic hemostasis	19 (mean)
Mohapatra S. et al., 2023 [49]	Case Report	1/0	1/1	0/1	Z-POEM	N/A	Perforation with abscess (1)	-	Endoscopic	Perforation: stent, Xtack, EVT	2
Norton B. et al., 2024 Cohort A [50]	Comparative retrospective single-center cohort series	25/15	45/45	18/27	Z-POEM	6.8 (3)	Leak (1), pneumonia (2) **^§^**	-	Conservative	No	6 (median)
Norton B. et al., 2024 Cohort B [50]	-	21/10	31/31	7/24	FESD	6.7 (2)	Perforation (1), abscess (1), pneumonia (1)	-	Surgery for drainage	No	5 (median)
Provenzano L. et al., 2025 [51]	Comparative matched multicenter retrospective cohort study	26/0	26/26	6/20	Z-POEM	15.4 (4)	Leak (1), emphysema (3)	-	Conservative	No	66 (median)
Rudler F. et al., 2023 [52]	Comparative retrospective multicenter cohort study	40/4	44/44	16/28	FESD	13 (6)	Bleeding (1), perforations (5)	-	Endoscopic	Perforation: clip TTS, stent	10.5 (median)
Rwigema J.C. et al., 2025 Cohort A [53]	Comparative retrospective single-center cohort series	2/2	4/4	1/3	Z-POEM	0	None	-	-	-	4 (median)
Rwigema J.C. et al., 2025 Cohort B [53]	-	6/3	9/9	3/6	Z-POEM	11 (1)	Perforation with fistula (1)	-	Endoscopic	Perforation: endoscopic closure	3 (median)
Rwigema J.C. et al., 2025 Cohort C [53]	-	58/4	62/62	28/34	Z-POEM	8.1 (5)	Leaks (2), perforation (2), abscess (1), mediastinitis (1), emphysema (1)	-	Surgery for drainage	No	1 (median)
Sanaei O. et al.,2020 [54]	Multicenter retrospective cohort study	32/32	32/32	13/19	Z-POEM	12.5 (4)	Mucosal tears (2), leaks (2)	Moderate	Endoscopic	Mucosal tears: clip TTS	5.5 (median)
Sarkis Y. et al., 2025 Cohort A [55]	Comparative retrospective single-center cohort series	18/3	21/21	12/9	FESD	28.6 (6)	Throat pain (1), atrial fibrillation (1), pneumomediastinum (2), perforation (3)	I-II-III-IV	Surgery for perforation	No	30 (median)
Sarkis Y. et al., 2025 Cohort B [55]	-	30/9	39/39	15/24	Z-POEM	5.1% (2)	Perforation (2)	III-IV	Endoscopic	Perforation: stent	12 (median)
Aden A. et al., 2023 [56]	Retrospective single-center cohort series	50/15	84/75	37/38	FESD	34.5 (29)	Bleeding (16), pneumonia (3), abscess (1), perforations (9), emphysema (2)	-	Surgery for drainage	Bleeding: thermal, clip TTS, injective hemostasis; perforations: stent, endoscopic suturing	13.2 (mean)
Antonello A. et al., 2015 Cohort A [57]	Comparative matched multicenter retrospective cohort study	25/25	25/25	5/20	FESD	8.8 (2)	Perforation (1), bleeding (1)	Mild	Endoscopic	Bleeding: thermal hemostasis (Coagrasper)	18 (median)
Antonello A. et al., 2015 Cohort B [57]	-	34/0	34/34	12/22	FESD	8.8 (3)	Perforation (1), bleeding (2)	Mild	Endoscopic	-	18 (median)
Buyruk A.M. et al., 2024 [58]	Retrospective single-center cohort series	19/1	20/20	13/7	Z-POEM	20 (4)	Emphysema (4)	Mild	Conservative	No	10 (median)
Diehl D.L. et al., 2022 [59]	Retrospective single-center cohort series	23/7	30/30	19/11	FESD	23.3 (7)	Perforation (1), throat pain (6)	Mild and moderate	Conservative	No	-
Dişibeyaz S. et al., 2017 [60]	Retrospective single-center cohort series	17/0	17/17	NR	FESD	17.6 (3)	Perforation (1), bleeding (2)	-	Endoscopic	Bleeding: clip TTS, thermal (heater probe) and injective hemostasis	47.7 (mean)
Economopoulos K.P. et al., 2025 [61]	Retrospective single-center cohort series	27/5	32/32	12/20	Z-POEM	3.1 (1)	Atrial fibrillation (1)	-	Conservative	No	10.8 (mean)
Juin C. et al., 2020 [62]	Retrospective single-center cohort series	13/6	19/19	9/10	FESD	10 (2)	Pneumonia (1), atrial fibrillation (1)	Mild and Severe	Conservative	No	9 (mean)
Kahaleh M. et al., 2022 Cohort A [63]	Comparative retrospective multicenter cohort study	49/0	49/49	NR	FESD	30.6 (15)	Bleeding (5), leaks (4), nausea/vomiting (3), bradycardia (1), pneumonia (1)	-	-	-	7.9 (mean)
Kahaleh M. et al., 2022 Cohort B [63]	-	52/0	52/52	NR	Z-POEM	9.6 (5)	Nausea/vomiting (2), bilateral pneumothorax (1), bleeding (2)	-	-	-	3.4 (mean)
Kenarli K. et al., 2025 [64]	Retrospective single-center cohort series	15/0	16/15	5/10	FESD	25% (4)	Bleeding (3), cervical inflammation (1)	Mild and Severe	Endoscopic	Bleeding: thermal hemostasis (APC)	24 (median)
Klingler M.J. et al., 2021 Cohort A [65]	Comparative retrospective single-center cohort series	7/0	7/7	1/6	FESD	28.6% (2)	Perforation (1), emphysema (1)	Moderate	Conservative	No	34.7 (median)
Klingler M.J. et al., 2021 Cohort B [65]	-	19/0	19/19	6/13	Z-POEM	15.8% (3)	Perforation (2), mediastinitis (1), abscess (1)	Moderate and Severe	Surgery for drainage	Abscess: endoscopic drainage and closure	9.5 (median)
Manzeneder J. et al., 2021 [66]	Retrospective single-center cohort series	100/0	126/100	36/64	FESD	19.8% (25)	Bleeding (16), perforation (5), chest pain (3), tachycardia (1)	Mild and moderate	Endoscopic	Bleeding: clip TTS, thermal (SB knife or Coagrasper) and injective hemostasis	41 (median)
Mavrogenis G. et al., 2024 [67]	Retrospective single-center cohort series	19/1	20/20	8/12	Z-POEM	5% (1)	Aspiration pneumonia (1)	-	Endoscopic	Clip removal due to closure of esophagus	18.5 (mean)
Mittal C. et al., 2021 Cohort A [68]	Comparative multicenter retrospective cohort study	137/0	137/137	NR	FESD	6.6% (9)	Perforation (4), bleeding (1), pain and dysphagia (4)	-	Endoscopic	Perforation: OTSC, clip TTS; dysphagia: clip removal	5.7 (mean)
Mittal C. et al., 2021 Cohort B [68]		24/0	24/24	NR	POES	16.7% (4)	Perforation (3), pain and dysphagia (1)	-	Endoscopic	Perforation: OTSC, clip TTS; dysphagia: clip removal	5.7 (mean)
Nabi Z. et al., 2019 [69]	Prospective single-center consecutive case series	5/2	7/7	2/5	FESD	42.9% (3)	Bleeding (3)	Minor	Endoscopic	Bleeding: thermal hemostasis (Knife)	-
Norton B. et al., 2025 [70]	Multicenter retrospective cohort study	38/23	61/61	23/38	Z-POEM	8.2% (5)	Leak (1), pneumoniae (2), refeeding syndrome (2)	II	Conservative	No	9 (median)
Pop A. et al., 2018 [71]	Retrospective single-center cohort series	31/0	31/31	14/17	FESD	9.67% (3)	Bleeding (3)	-	Endoscopic	Bleeding: clip TTS, injective hemostasis	-
Ramchandani M. et al., 2013 [72]	Prospective single-center consecutive case series	3/0	3/3	1/2	FESD	33.3% (1)	Bleeding (1)	Minor	Endoscopic	Bleeding: thermal hemostasis (Knife)	-
Che S.Y.W. et al., 2023 [73]	Retrospective single-center cohort series	54/0	55/54	22/32	Z-POEM	9.2% (5)	Leak (3), dysphagia (1), pneumonia (1)	-	Conservative	No	2 (median)
Steinway S. et al., 2023 [74]	Multicenter retrospective cohort study	68/21	89/89	38/51	Z-POEM	8.9% (8)	Leaks (3), bleeding (3), pneumonia (1), pneumomediastinum (1)	Mild and moderate	Endoscopic	Endoscopic closure and hemostasis	37 (mean)

Legend: AEs, adverse events; APC, argon plasma coagulation; EVT, endoscopic vacuum therapy; FESD, flexible endoscopic septotomy; OTSC, over-the-scope clip; POES; peroral endoscopic septotomy; SB Knife, Stag Beetle knife; SING, submucosal injection-guided septomyotomy; TTS, through-the-scope; Z-POEM, Zenker’s peroral endoscopic myotomy. Notes: -, not reported value; ^§^, fatal outcome. Procedural AE rates are calculated using the total number of distinct endoscopic procedures as denominator. Single-case reports (*n* = 7) and overlapping multicenter cohort iterations were retained exclusively for qualitative complication and rescue mapping and excluded from pooled quantitative incidence calculations. Discrepancies between total AE counts and itemized complication sums in select series reflect patients experiencing concomitant or sequential AEs.

**Table 2:** Cumulative rates and subgroup distribution of adverse events.

Incidence	Bleeding	Perforation	Leak	Emphysema	Abscess	Mediastinitis	Pneumonia	Other AEs
Overall, % (*n*)	4.2 (137)	3.2 (103)	0.6 (21)	1.3 (43)	0.2 (6)	0.1 (4)	0.5 (17)	2.2 (73)
FESD, % (*n*)	5.9 (124)	3.9 (82)	0.2 (4)	1.1 (24)	0.2 (4)	0.1 (2)	0.4 (9)	2.4 (50)
Tunneling techniques, % (*n*)	1.1 (13)	1.8 (21)	1.5 (17)	1.7 (19)	0.2 (2)	0.2 (2)	0.7 (8)	2.0 (23)
Overall cohorts, *n*	37/64	41/64	14/64	16/64	6/64	4/64	12/64	25/64
FESD cohorts, *n*	31/39	26/39	3/39	9/39	4/39	2/39	7/39	14/39
Tunneling cohorts, *n*	6/25	15/25	11/25	7/25	2/25	2/25	5/25	11/25

Legend: AEs, adverse events; FESD, flexible endoscopic septotomy. Note: Denominators represent distinct endoscopic procedures (Total: 3266; FESD: 2119; Tunneling: 1147). Single-case reports (*n* = 7) and overlapping cohorts were excluded from quantitative pooled incidence.

## Data Availability

The raw data supporting the conclusions of this article will be made available by the authors upon reasonable request.

## Supplementary Materials

The following supporting information can be downloaded at: https://www.mdpi.com/article/10.3390/medicina62102029/s1, Supplementary Material S1: Search Strings; Supplementary Material S2: PRISMA 2020 Checklist; Methodological Quality Assessment.

## Abbreviations

The following abbreviations are used in this manuscript:

AE	Adverse event
AGREE	Appraisal of Guidelines for Research & Evaluation
ASA	American Society of Anesthesiologists
ASGE	American Society for Gastrointestinal Endoscopy
CO_2_	Carbon dioxide
CP	Cricopharyngeal
CPAP	Continuous positive airway pressure
EGD	Esophagogastroduodenoscopy
ESGE	European Society of Gastrointestinal Endoscopy
EVT	Endoscopic vacuum therapy
FESD	Flexible endoscopic septum division
MINORS	Methodological Index for Non-Randomized Studies
NiZ-POEM	Non-tunneling Z-POEM
OZ-POEM	Open Z-POEM
POED	Peroral endoscopic diverticulotomy
POES	Peroral endoscopic septotomy
PRISMA	Preferred Reporting Items for Systematic Reviews and Meta-Analyses
SING	Submucosal injection guided septomyotomy
TILS	Transverse incision and longitudinal septostomy
ZD	Zenker’s diverticulum
Z-POEM	Zenker’s peroral endoscopic myotomy
