# Supplementary material for: Safety Profile and Adverse Event Management in Flexible Endoscopic Treatment of Zenker’s Diverticulum: A Systematic Review
Source: Medicina (Kaunas). 2026 Oct 20;62(10):2029. doi: 10.3390/medicina62102029 (PMC13707217; doi:10.3390/medicina62102029)
Supplement: Supplementary file 1 [file medicina-62-02029-s001.zip › Supplementary Material S1-search strings.pdf]

Supplementary Material S1: Search Strings

Bibliographic search

Bibliographic search strategies were performed to 30 June 2026 using the following search strategies

<u>*Search string (Pubmed)*</u>:

("Zenker Diverticulum"[Mesh] OR "Zenker diverticulum"[tiab] OR "Zenker's diverticulum"[tiab] OR "pharyngoesophageal diverticulum"[tiab] OR "pharyngeal pouch"[tiab] OR "hypopharyngeal diverticulum"[tiab]) AND ("Endoscopy"[Mesh] OR endoscop*[tiab] OR "peroral endoscopic myotomy"[tiab] OR POEM[tiab] OR "Z-POEM"[tiab] OR "zenker peroral endoscopic myotomy"[tiab] OR septotom*[tiab] OR septomyotom*[tiab] OR diverticulotom*[tiab] OR stapl*[tiab]) AND ("Recurrence"[Mesh] OR "Treatment Failure"[Mesh] OR "Postoperative Complications"[Mesh] OR recurren*[tiab] OR relapse*[tiab] OR failure*[tiab] OR redo[tiab] OR "re-do"[tiab] OR retreat*[tiab] OR reintervention*[tiab] OR "re-intervention"[tiab] OR reoperation*[tiab] OR complication*[tiab] OR "adverse event*"[tiab] OR perforat*[tiab] OR bleed*[tiab] OR emphysema[tiab] OR leak*[tiab] OR fistula*[tiab] OR mediastinitis[tiab] OR infection*[tiab] OR pneumonia*[tiab] OR dysphagia*[tiab] OR "surgical rescue"[tiab] OR "conversion to surgery"[tiab])
– 666 results

<u>*Search string (EMBASE)*</u>:

('zenker diverticulum'/exp OR 'zenker diverticulum':ti,ab OR 'pharyngoesophageal diverticulum':ti,ab OR 'pharyngeal pouch':ti,ab OR 'hypopharyngeal diverticulum':ti,ab) AND ('endoscopy'/exp OR endoscop*:ti,ab OR 'peroral endoscopic myotomy':ti,ab OR poem:ti,ab OR 'z-poem':ti,ab OR 'zenker peroral endoscopic myotomy':ti,ab OR septotom*:ti,ab OR septomyotom*:ti,ab OR diverticulotom*:ti,ab OR stapl*:ti,ab) AND ('procedure complication'/exp OR 'treatment failure'/exp OR 'disease recurrence'/exp OR 'reoperation'/exp OR recurren*:ti,ab OR relapse*:ti,ab OR failure*:ti,ab OR redo:ti,ab OR 're-do':ti,ab OR retreat*:ti,ab OR reintervention*:ti,ab OR 're-intervention':ti,ab OR reoperation*:ti,ab OR complication*:ti,ab OR 'adverse event*':ti,ab OR perforat*:ti,ab OR bleed*:ti,ab OR emphysema:ti,ab OR leak*:ti,ab OR fistula*:ti,ab OR mediastinitis:ti,ab OR infection*:ti,ab OR pneumonia*:ti,ab OR dysphagia*:ti,ab OR 'surgical rescue':ti,ab OR 'conversion to surgery':ti,ab)
– 1,273 results

<u>*Search string (Scopus)*</u>:
("Zenker diverticulum" OR "Zenker's diverticulum" OR "pharyngoesophageal diverticulum" OR "pharyngeal pouch" OR "hypopharyngeal diverticulum") AND TITLE-ABS-KEY(endoscop* OR "peroral endoscopic myotomy" OR POEM OR "Z-POEM" OR "Zenker peroral endoscopic myotomy" OR septotom* OR septomyotom* OR diverticulotom* OR stapl*) AND TITLE-ABS-KEY(recurren* OR relapse* OR failure* OR redo OR "re-do" OR retreat* OR reintervention* OR "re-intervention" OR reoperation* OR complication* OR "adverse event*" OR perforat* OR bleed* OR emphysema OR leak* OR fistula* OR mediastinitis OR infection* OR pneumonia* OR dysphagia* OR "surgical rescue" OR "conversion to surgery")
– 1,077 results
